# Economic burden of patients with post-surgical chronic and transient hypoparathyroidism in the United States examined using insurance claims data

**DOI:** 10.1186/s13023-024-03155-4

**Published:** 2024-04-18

**Authors:** Kathleen L Deering, Niccole J Larsen, Patrick Loustau, Blandine Weiss, Soraya Allas, Michael D Culler, Qing Harshaw, Deborah M. Mitchell

**Affiliations:** 1 EPI-Q, Inc, Chicago, IL USA; 2 Amolyt Pharma, Cambridge, MA USA; 3 Amolyt Pharma, Ecully, France; 4 Massachusetts General Hospital, Boston, MA USA

**Keywords:** Chronic hypoparathyroidism, Economic burden, Healthcare burden, Costs, Claims analysis

## Abstract

**Background:**

Hypoparathyroidism (HP) is a rare endocrine disease commonly caused by the removal or damage of parathyroid glands during surgery and resulting in transient (tHP) or chronic (cHP) disease. cHP is associated with multiple complications and comorbid conditions; however, the economic burden has not been well characterized. The objective of this study was to evaluate the healthcare resource utilization (HCRU) and costs associated with post-surgical cHP, using tHP as a reference.

**Methods:**

This analysis of a US claims database included patients with both an insurance claim for HP and thyroid/neck surgery between October 2014 and December 2019. cHP was defined as an HP claim ≥ 6 months following surgery and tHP was defined as only one HP claim < 6 months following surgery. The cHP index date was the first HP diagnosis claim following their qualifying surgery claim, whereas the tHP index date was the last HP diagnosis claim following the qualifying surgery claim. Patients were continuously enrolled at least 1 year pre- and post-index. Patients’ demographic and clinical characteristics, all-cause HCRU, and costs were descriptively analyzed. Total all-cause costs were calculated as the sum of payments for hospitalizations, emergency department, office/clinic visits, and pharmacy.

**Results:**

A total of 1,406 cHP and 773 tHP patients met inclusion criteria. The average age (52.1 years cHP, 53.5 years tHP) and representation of females (83.2% cHP, 81.2% tHP) were similar for both groups. Neck dissection surgery was more prevalent in cHP patients (23.6%) than tHP patients (5.3%). During the 1–2 year follow-up period, cHP patients had a higher prevalence of inpatient admissions (17.4%), and emergency visits (26.0%) than the reference group -tHP patients (14.4% and 21.4% respectively). Among those with a hospitalization, the average number of hospitalizations was 1.5-fold higher for cHP patients. cHP patients also saw more specialists, including endocrinologists (28.7% cHP, 15.8% tHP), cardiologists (16.7% cHP, 9.7% tHP), and nephrologists (4.6% cHP, 3.3% tHP).

**Conclusion:**

This study demonstrates the increased healthcare burden of cHP on the healthcare system in contrast to patients with tHP. Effective treatment options are needed to minimize the additional resources utilized by patients whose HP becomes chronic.

**Supplementary Information:**

The online version contains supplementary material available at 10.1186/s13023-024-03155-4.

## Introduction

Hypoparathyroidism (HP) is a rare disease diagnosed by the presence of both hypocalcemia and insufficient levels of serum parathyroid hormone (PTH) [1–3]. PTH hormone plays a crucial role in regulating calcium, vitamin D, and phosphorus levels in the body. The clinical manifestations of HP stem from hypocalcemia-related alterations in neuromuscular, cognitive, and cardiac function [4–6]. Common symptoms include cognitive dysfunction such as fatigue and “brain fog,” paresthesia, cramps, tetany and acute manifestation of seizures, bronchospasm, laryngospasm, and cardiac rhythm disturbances [6]. HP is typically caused by removal of or damage to the parathyroid glands during surgery but can also arise secondary to other conditions including autoimmune disease, congenital conditions, and idiopathic causes [4, 7, 8]. 

At the time this study was conducted, HP resulting from surgery was considered transient if the biochemical abnormalities resolved within 6 months of surgery and chronic if abnormalities persisted 6 months after the surgery [9]. In 2022, an international task force on HP published new guidelines defining HP as permanent (chronic) if it persists > 12 months after surgery [2]. The key distinction is the duration and reversibility of the condition, with transient HP (tHP) being temporary, whereas chronic HP (cHP) is a long-standing, often permanent condition [4–6]. While in recent years, there has been a surge in the interest in and published articles on cHP [10, 11], there is a paucity of literature describing how the chronicity of the disease and its treatment impacts outcomes, quality of life, and healthcare burden of patients suffering from cHP differs from those with tHP.

Calcium supplementation and activated forms of vitamin D remain the mainstays for the treatment and management of HP [2, 10]. To date, the only FDA-approved therapy is full-length recombinant human PTH1–84; however, it was withdrawn from the US market in September 2019 and available to patients only through a Special Use Program. In October 2023 the manufacturer announced the end of production in 2024 due to unresolved manufacturing issues that are specific to the product [12]. Teriparatide (human PTH1-34, the N-terminal fragment of human PTH indicated for the treatment of osteoporosis), is used off-label for the treatment of cHP in some cases [2, 10, 13–16]. The recommended goal for the treatment of HP is maintenance of serum calcium level within the low-normal range, to limit hypercalcemia and hypercalciuria, while avoiding symptomatic hypocalcemia [2, 7, 9, 16]. Additional treatment considerations may be made regarding the preservation of bone health, especially in cHP [17, 18]. 

Long-term complications of cHP and use of activated vitamin D and calcium supplementation as treatment of cHP may contribute to increased renal (e.g., nephrolithiasis, nephrocalcinosis, and decreased renal function) and cardiovascular (e.g., cardiomyopathy, congestive heart failure, and arrhythmia) complications [4, 19–22]. Other complications associated with cHP and the use of activated vitamin D and calcium supplementation include: soft tissue calcifications, basal ganglia calcifications, cataracts, increased frequency of infectious diseases and neuropsychiatric disorders [4, 18, 21]. Additionally, a significant number of post-menopausal patients with cHP have osteopenia or osteoporosis and certain HP populations including post-menopausal women may be at risk for vertebral fractures [17, 18, 23]. Recent studies have also shown higher mortality rates and decreased quality of life in patients with cHP [4, 21, 24–28]. 

The majority of the epidemiologic and economic studies conducted on cHP thus far have been outside of the United States [10, 11]. This study aims to improve our knowledge of the financial burden to patients and healthcare systems through an analysis of United States claims data to evaluate the healthcare resource utilization (HCRU) in patients with post-surgical cHP.

## Methods

### Data source

This retrospective cohort study was an analysis of health insurance claims during the period ranging from October 1, 2014 to December 31, 2019 (Supplementary Fig. 1). Anonymized claims data were obtained from a HealthVerity Closed Payer Claim Medical and Pharmacy Database (Private Source 20) that included a total of 130 million members who were enrolled in Commercial, Medicare Advantage, or Medicaid plans across 150 payers. The HealthVerity Private Source 20 database includes people from all regions of the United States and has been used in numerous studies [29]. Information found in this database includes insurance enrollment dates, demographic characteristics, outpatient and inpatient services and diagnoses, procedures, prescription drugs, and other services. The cost data represents payments of adjudicated medical and pharmacy claims. This study was considered exempt under 45 CFR § 46.104(d) [4].

### Study population

We identified post-surgical patients with diagnosis claims for HP (International Classification of Diseases (ICD) 9/10 codes: E20.0, E20.8, E20.9, E89.2, 252.1) and grouped them into chronic and transient HP cohorts (see Supplementary Table 1 for surgical and HP diagnosis codes). The criteria for cHP were adapted from Powers et al [3] and defined under the guidance of clinicians experienced in treating cHP. All patients were continuously enrolled in their insurance plan at least 1 year pre- and post-index to evaluate outcomes for a minimum of 1 year pre- and post-index.

The cHP cohort required patients to have a claim for parathyroidectomy, complete or partial thyroidectomy, or neck dissection followed by a claim with a diagnosis of HP. Patients with a diagnosis of HP 6–15 months following their qualifying surgical claim, no HP diagnosis claim prior to surgery, and a second HP diagnosis claim at any subsequent time point were categorized as cHP patients (Supplementary Fig. 1). The index date for the cHP group was defined as the date of the first qualifying HP diagnosis claim.

Patients with a diagnosis of HP within 6 months following the procedure, with no HP diagnosis claim before the procedure nor any subsequent claims 6 or more months after the procedure were categorized as tHP patients (Supplementary Fig. 1). The tHP group served as a reference group, and the date of the last HP diagnosis claim following the procedure was their index date.

### Measures

Patient demographics included age, sex, geographic location, and insurance type at index. Baseline clinical characteristics were composed of the Quan-Charlson Comorbidity Index (CCI) [30] and selected conditions associated with HP (e.g., calcium and bone disorders, gastrointestinal disorders, neuropsychiatric conditions). Baseline demographics and clinical characteristics were analyzed for one year prior to the surgical procedure date.

Outcome measures included were HCRU and costs. These were identified through associated ICD 9/10 diagnostic or procedure codes, Healthcare Common Procedure Coding System (HCPCS), or Common Procedural Technology (CPT) codes. All-cause HCRU and costs were reported for inpatient and outpatient hospitalizations, emergency department visits, office/clinic visits, and pharmacy use. HCRU patterns and costs were analyzed at index to 1-year post-index and year 1 through year 2 post-index.

### Statistical analysis

This was intended to be a descriptive study of the cHP population. The authors determined it was important to provide reference for the cHP findings and created the tHP group in a post-hoc analysis. Therefore, statistical comparisons were not conducted between the groups and all data reported are unadjusted for duration of follow-up, inflation costs, etc. Descriptive statistics were used to report demographics, clinical characteristics, and HCRU patterns and costs. Means and standard deviations (SD) were reported for continuous variables, and frequencies and percentages were reported for categorical variables. Missing data was evaluated and reported as “unknown” for any patient demographic variables. All patients were included in the cost analyses, regardless of whether costs were reported or incurred. All analyses were conducted using SAS version 9.4 (Cary, North Carolina, USA).

## Results

### Study population

Of the 43,640 patients with a diagnosis claim for HP in the claims database during the study period of October 1, 2014 to December 31, 2019, a total of 2,179 patients met eligibility criteria and were identified as having post-surgical hypoparathyroidism. Of these, 1,406 were classified as cHP and 773 as tHP (reference group) (Supplementary Figs. 2 and 3). The mean time to the qualifying HP diagnosis after neck surgery was 8.7 (SD 2.3) months for the cHP cohort and 2.0 (SD 1.7) months for the tHP cohort. The most common neck surgery for both the cHP and tHP cohorts was thyroidectomy (50.2% and 57.9%, respectively). Nearly a quarter of both cHP and tHP cohorts had a parathyroidectomy (26.2% and 23%, respectively). Neck dissection was more prevalent in the cHP cohort (23.6%) than in the tHP cohort (5.1%) (Table 1).

**Table 1:** Baseline characteristics

	cHP Cohort (*N* = 1,406)	tHP Cohort (*N* = 773)
**Female, n (%)**	1,170 (83.2%)	624 (81.2%)
**Age (Years), Mean (SD)**	52.1 (16.4)	53.5 (14.9)
≤ 40	353 (25.1%)	167 (22.2%)
41–50	292 (20.8%)	132 (17.6%)
51–64	443 (31.5%)	292 (37.8%)
> 65	318 (22.6%)	182 (23.5%)
**Charlson Comorbidity Index, Mean (SD)**	4.1 (3.6)	3.3 (3.3)
**Charlson Comorbidity Index, n (%)**		
0	223 (15.9%)	179 (23.2%)
1	141 (10.0%)	87 (11.3%)
2	273 (19.4%)	166 (21.5%)
>=3	769 (54.7%)	341 (44.1%)
**Insurance type, n (%)**		
Commercial	743 (52.8%)	452 (58.5%)
Medicaid	397 (28.3%)	204 (26.4%)
Medicare Advantage	220 (15.7%)	87 (11.3%)
Unknown	42 (2.9%)	30 (3.0%)
**Procedures, n (%)**		
Parathyroidectomy	368 (26.2%)	286 (37.0%)
Neck dissection	332 (23.6%)	39 (5.1%)
Thyroidectomy	706 (50.2%)	448 (57.9%)
**Time between surgery and HP claim that qualified the patient for eligibility (Months), Mean (SD)**	8.7 (2.3)	2.0 (1.7)
**Patients with HP Code before surgery**	115 (8.2%)	n/a
**Region, n (%)**		
Midwest	875 (21.2%)	208 (26.9%)
Northeast	990 (24.0%)	168 (21.7%)
South	1,002 (24.3%)	235 (30.4%)
West	1,158 (28.1%)	151 (19.5%)
Alaska	1 (0.1%)	0 (0.0%)
Hawaii	19 (0.5%)	1 (0.1%)
Puerto Rico	72 (1.7%)	10 (1.3%)

cHP: Chronic Hypoparathyroidism; HP: Hypoparathyroidism; SD: Standard Deviation; tHP: Transient Hypoparathyroidism

Demographic information and baseline characteristics of patients are summarized in Table 1. Approximately 80% of patients with HP included in this analysis were female with a mean (SD) age of 52.1 (16.4) years for the cHP cohort and 53.5 (14.9) years for the tHP cohort. The majority of patients in the cHP and tHP cohorts had Commercial insurance (52.8% and 58.5%). Nearly 30% of patients had Medicaid insurance (28.3% cHP; 26.4% tHP). Medicare Advantage covered 15.7% of patients in the cHP cohort and 11.3% in the tHP cohort. The mean (SD) CCI score for patients with cHP and tHP cohorts was 4.1 (3.6) and 3.3 (3.3), respectively. Before surgery, ≥ 5% difference was observed between the cHP and tHP cohorts for the following conditions: hyper- and hypocalcemia, any malignancies, and in particular thyroid cancer (Table 2). Rates of other comorbid conditions were generally similar across both cohorts.

**Table 2:** Baseline Comorbidities, 1 year before Surgery

	cHP Cohort *N* = 1,069^a^	tHP Cohort *N* = 773
**Cardiovascular and metabolic disorders**		
Arrhythmias	195 (18.2%)	113 (14.6%)
Congestive heart failure	60 (5.6%)	33 (4.3%)
Diabetes	225 (21.0%)	175 (22.6%)
Hypertension	485 (45.4%)	331 (42.8%)
**Central Nervous System**		
Basal ganglia calcification	0 (0.0%)	0 (0.0%)
Peripheral Neuropathy	25 (2.3%)	9 (1.2%)
Seizures and convulsions	21 (2.0%)	10 (1.3%)
**Laboratory Imbalances**		
Hypercalcemia	142 (13.3%)	144 (18.6%)
Hypercalciuria	9 (0.8%)	5 (0.6%)
Hypocalcemia	77 (7.2%)	8 (1.0%)
**Malignancy**		
Any Malignancy	457 (42.8%)	265 (34.3%)
Thyroid Cancer	379 (35.5%)	212 (27.4%)
**Musculoskeletal**		
Fractures	32 (3.0%)	19 (2.5%)
Osteoporosis	95 (8.9%)	50 (6.5%)
**Neuropsychiatric**		
Anxiety	236 (22.1%)	165 (21.3%)
Cognitive impairment	27 (2.5%)	15 (1.9%)
Dementia	10 (0.9%)	6 (0.8%)
Depressive disorders	202 (18.9%)	135 (17.5)
Sleep-wake disorders	190 (17.8%)	115 (14.9%)
**Other**		
Cataract formation	0 (0.0%)	0 (0.0%)
Tetany	2 (0.2%)	1 (0.1%)
**Renal Disease**		
CKD (Stage 1–4, unspecified)	87 (8.1%)	54 (7.0%)
CKD Stage 5, ESRD and failure	44 (4.1%)	31 (4.0%)
Diabetic Nephropathy	87 (8.1%)	59 (7.6%)
Nephrolithiasis/renal stones	65 (6.1%)	54 (7.0%)

^a^Comorbidities are based on claims from the 1-year pre-index period and not all patients had a full 1-year of data

CHF: Congestive Heart Failure; cHP: Chronic Hypoparathyroidism; CKD: Chronic Kidney Disease; CNS: Central Nervous System; ESRD: End Stage Renal Disease; tHP: Transient Hypoparathyroidism

### All-cause HCRU

**Fig. 1:**
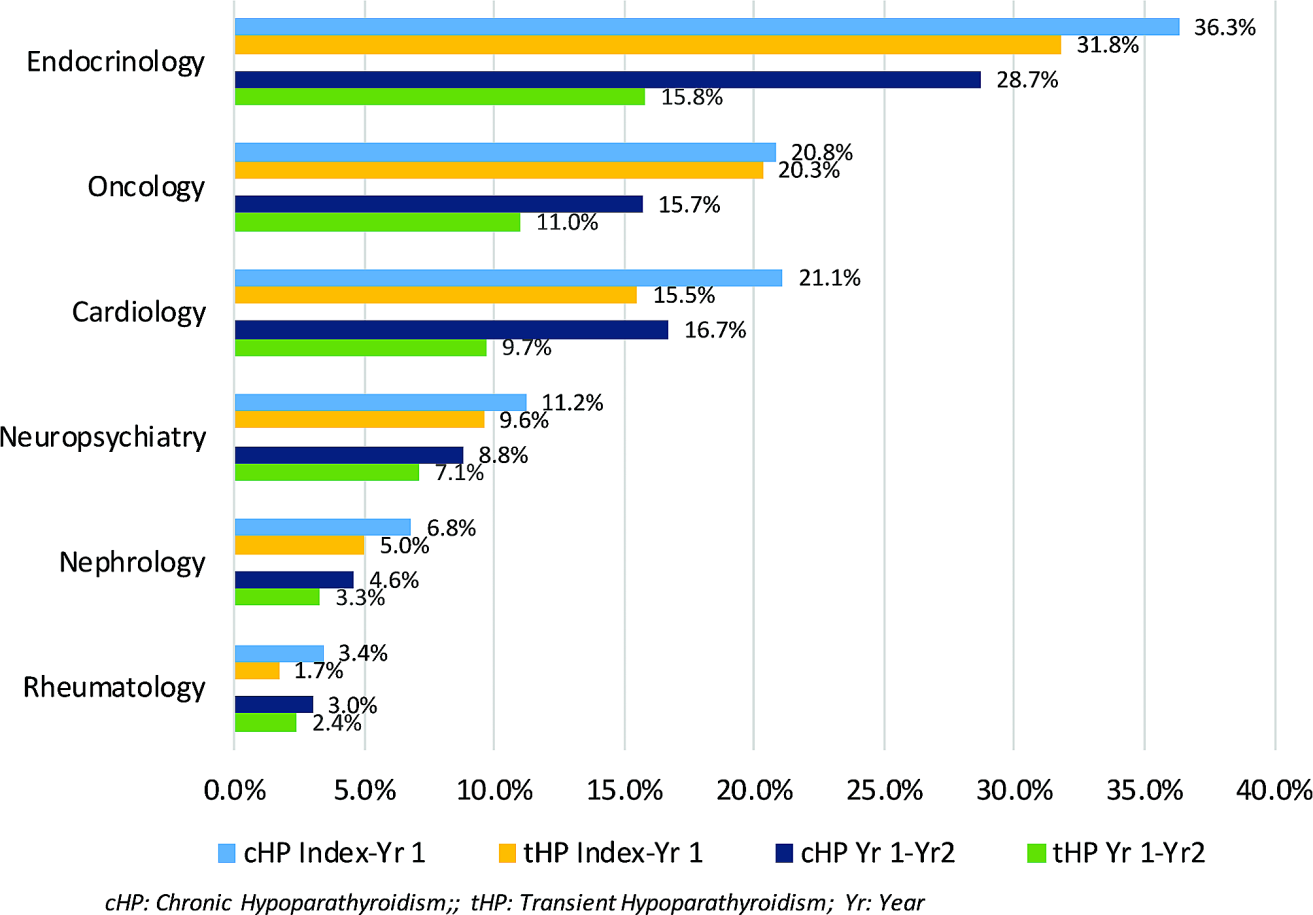
All-cause provider resource utilization

In the year post-index, the proportion of patients with all-cause emergency department visits was 32.7% in the cHP cohort and 29.1% in the tHP cohort, while the proportion of patients with office/clinic visits, outpatient hospitalizations, and inpatient hospitalizations were nearly equal between the cohorts (Table 3). Despite similar proportions and frequencies of office/clinic visits, patients in the cHP cohort had more specialty provider visits during the first-year post-index. The specialties evaluated included endocrinology (36.3% cHP, 31.8% tHP), cardiology (21.1% cHP, 15.5% tHP), oncology (20.8% cHP, 20.3% tHP), neuropsychiatry (11.2% cHP, 9.6% tHP), nephrology (6.8% cHP, 5.0% tHP), and rheumatology (3.4% cHP, 1.7% tHP) (Fig. 1).

**Table 3:** All-Cause HCRU

	cHP Cohort			tHP Cohort		
	Follow-up Index-1 Year *N* = 1,184	Follow-up Year 1–2 *N* = 726	Follow-up Index-1 Year *N* = 773		Follow-up Year 1–2 *N* = 575	
Percentage of Patients with ≥ 1 visit, n (%)						
Inpatient Hospital	303 (25.6%)	126 (17.4%)	194 (25.1%)		83 (14.4%)	
Outpatient Hospital	1,021 (86.2%)	576 (79.3%)	667 (86.3%)		431 (75.0%)	
Emergency Department	387 (32.7%)	189 (26.0%)	225 (29.1%)		123 (21.4%)	
Office/Clinic Visit	1,139 (96.2%)	672 (92.6%)	748 (96.8%)		504 (87.7%)	
Urgent Care Facility	96 (8.1%)	54 (7.4%)	60 (7.8%)		52 (9.0%)	
**Number of visits for the total population, Mean (SD)**						
Inpatient Hospital	2.2 (8.9)	1.4 (6.2)	1.7 (6.4)		0.8 (3.4)	
Outpatient Hospital	9.6 (19.0)	6.6 (11.1)	8.3 (20.8)		6.2 (20.7)	
Emergency Department	1.1 (3.1)	0.8 (2.2)	0.7 (2.0)		0.5 (1.6)	
Office/Clinic Visit	16.7 (18.9)	15.2 (20.3)	16.0 (21.9)		12.5 (17.1)	
Urgent Care Facility	0.2 (0.6)	0.1 (0.5)	0.1 (0.7)		0.1 (0.5)	
**Number of visits of those who utilized, Mean (SD)** ^**a**^						
Inpatient Hospital	11.1 (20.1)	8.3 (11.8)	6.9 (11.3)		5.7 (7.4)	
Outpatient Hospital	8.8 (15.9)	8.3 (12.7)	9.6 (22.1)		8.3 (23.5)	
Emergency Department	3.3 (4.7)	3.0 (3.4)	2.5 (3.0)		2.4 (2.7)	
Office/Clinic Visit	17.4 (19.0)	16.4 (20.6)	16.5 (22.1)		14.3 (17.6)	
Urgent Care Facility	1.9 (1.3)	1.6 (1.2)	1.9 (1.7)		1.4 (0.8)	

^a^Denominators are based on the number of patients with ≥ 1 visit for the respective healthcare setting

cHP: Chronic Hypoparathyroidism; SD: Standard Deviation; tHP: Transient Hypoparathyroidism

Between year 1 to 2 post-index, the proportion of patients with HCRU was higher in the cHP cohort than in the tHP cohort (Table 3). The proportion of patients in the cHP cohort with an inpatient admission was 17.4% and 26.0% had an emergency visit. For the tHP cohort, 14.4% had an inpatient admission and 21.4% had an emergency visit. Among those who were hospitalized, the average number of hospitalizations in the cHP cohort was ~ 1.5 times greater than the tHP cohort. Additionally, patients in the cHP cohort continued to see a higher variety of specialty providers at a higher frequency in the year 1 to 2 post-index follow-up period (Fig. 1).

### All-cause healthcare resource costs

**Fig. 2:**
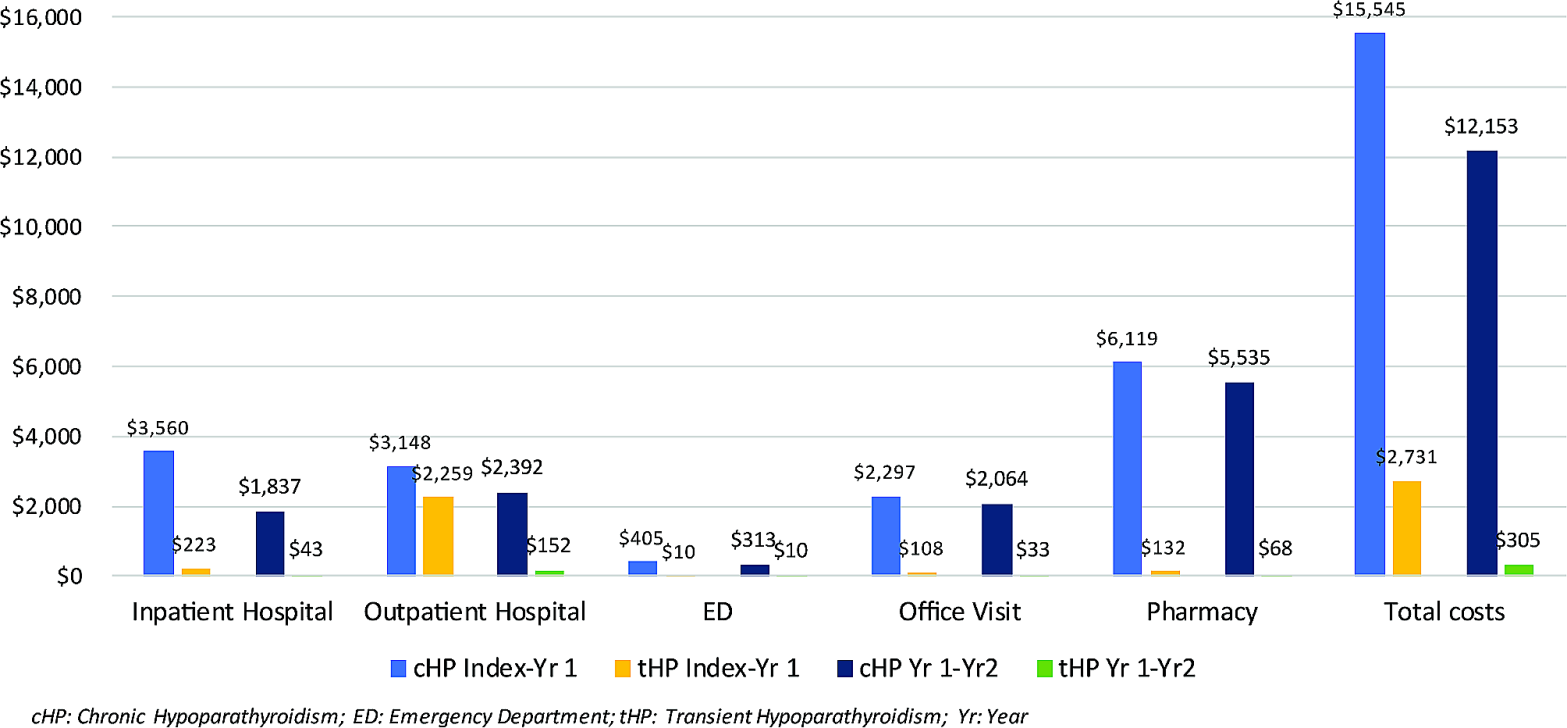
All-cause healthcare resource costs

The cHP cohort had nearly 5 times the mean (SD) unadjusted total all-cause costs than the tHP cohort during the first-year post-index: $15,545 ($27,902) and $2,731 ($27,677), respectively (Fig. 2). Inpatient hospitalizations, office/clinic visits, and pharmacy costs were drivers of the unadjusted cost differences. During 1-year post-index, the cHP cohort had nearly 15 times higher reported inpatient hospitalization costs, 40 times higher emergency department costs, and 20 times greater office/clinic visit costs than the tHP cohort. The largest difference was in the mean (SD) unadjusted pharmacy costs, with the cHP cohort incurring $6,119 ($18,155) compared to $132 ($909) for the tHP cohort.

Between the year 1 and 2 post-index period, the mean (SD) unadjusted total all-cause costs decreased for both groups; however, the cHP cohort still had costs 40 times higher than the tHP cohort: $12,153 ($22,341) versus $305 ($1,585), respectively (Fig. 2). The mean (SD) unadjusted all-cause inpatient costs were nearly 45 times higher in the cHP cohort than the tHP cohort during year 1 and 2 post-index: $1,837 ($6,564) versus $43 ($641). Office/clinic visits and pharmacy costs also remained higher for the cHP cohort during this period.

## Discussion

Using a database including claims from patients covered by Commercial, Medicare, and Medicaid plans and rigorous eligibility criteria for the identification of cHP patients, we find that patients with post-surgical cHP have substantially greater HCRU and cost burden than those with post-surgical tHP, serving as a reference group.

Throughout the study period, the cHP cohort experienced nearly double the number of all-cause emergency room visits and inpatient hospitalizations than the tHP cohort. Additionally, patients from the cHP cohort sought more specialist care during follow-up periods. The cHP cohort incurred approximately $12,153 in total all-cause costs compared to $305 for the tHP cohort between year 1 and 2 post-index, driven by hospitalizations and pharmacy costs. These costs are likely to be underestimated as healthcare costs were not reported for all plans that contribute to HealthVerity Closed Payer Claim Medical and Pharmacy Database (Private Source 20).

Patient demographics of the cHP cohort were consistent with previous reports in that the majority of cHP patients were female with mean age consistent with peri- or post-menopause [25, 31–33]. The baseline comorbidity burden of patients with cHP is also consistent with reports from previous studies [25, 31–34]. Patients from the cHP cohort had a slightly higher CCI score at baseline compared to those in the tHP cohort, presumably due to the higher rate of thyroid cancer, indicating they are at greater risk for both morbidity and mortality [30]. However, patients with cHP are also at higher risk of short-term and long-term complications such as renal impairment, cardiovascular and cerebrovascular disease, infection, and mental illness, all of which may contribute to higher HCRU and costs [4, 21, 27, 31, 35]. 

There have been very few studies evaluating the HRCU and cost burden of patients with cHP [25, 31, 35, 36]. In a multi-country chart review, it was reported that patients with HP not adequately controlled on standard of care therapy (calcium and activated vitamin D supplements) experienced substantial clinical burden of illness (e.g., persistent symptoms and multiple comorbidities) which resulted in higher HP-related HCRU compared to those who were adequately controlled [31]. The HP-related HCRU reported for not adequately controlled compared to adequately controlled included: hospitalizations (27.9% vs. 16.3%; *p* < 0.01), ER visits (47.7% vs. 38.5%; *p* < 0.05), and outpatient visits (89.5% vs. 87.1%; *p* = 0.49). It was also noted that the not adequately-controlled group had significantly higher rates of complications and comorbidities compared with patients with adequately-controlled cHP which may have contributed to the higher rate of HCRU.

High rates of complications, comorbidities and HCRU burden findings for patients with cHP in the current study are also supported by patient surveys [36, 37]. In the PARADOX study, 69% of patients with cHP reported complications and comorbidities and 79% reported HP-related hospitalization or ER visits in 1-year [36]. Additionally, a patient survey conducted in Germany reported 31% of patients with cHP had an outpatient visit and 5% had a hospitalization due to HP-related symptoms in a 12-month period [37]. In this study, 40% of patients also reported presenting at least once to the emergency department since their primary diagnosis because of severe hypocalcemic symptoms [37]. 

Finally, in a study presented at the European Congress of Endocrinology 2021, the clinical and economic burden of cHP on secondary care in England was evaluated among patients with post-surgical and non-surgical cHP compared with those of patients with hypothyroidism and those who underwent thyroid surgery, respectively [38]. The authors found complications, comorbidities, HCRU, and accrued annual inpatient and outpatient costs were higher in non-surgical and post-surgical cHP patients vs. their respective comparator groups. Additionally, it was reported that the main cost driver was renal complications across all cohorts.

### Limitations

Differences in study methodology and patient characteristics may account for the difference in HCRU reported in previous studies compared to the current study. Due to the nature of claims databases, our study was unable to evaluate disease control and other clinical characteristics which have been shown to impact HCRU and costs.

Common among database claims analysis studies, misinformation due to misclassification and miscoded conditions could have been significant for the current study. To reduce the issue of cHP misclassification, we required a minimum of two claims with an HP diagnosis. Powers, et al. (2013) described the high rates (66%) of physicians across several specialties self-reporting not utilizing the HP classification codes, and rather using classifications codes for hypocalcemia instead [3]. Therefore, our results may be an underrepresentation of the HP post-surgical populations.

Extrapolation of observations to the uninsured population and inability to assess the use of over-the-counter calcium supplementation which is the most common therapeutic option for patients with cHP, are also potential limitations of this study.

## Conclusion

This large claims database analysis provides essential insights on the HCRU and cost burden of the cHP population to the United States healthcare system. It was observed that the chronicity of HP appears to contribute to higher utilization of healthcare services and incurred healthcare costs compared to patients with transient disease. These findings suggest a need for improved management and treatment of cHP. Future studies are required to better understand the economic impact associated with cHP complications and comorbidities, and to investigate ways to reduce overall burden of disease and costs associated with cHP.

### Electronic supplementary material

Below is the link to the electronic supplementary material.

Supplementary Material 1

## Data Availability

The proprietary databases used for this study were made available to Amolyt Pharma and EPI-Q through a license that limits dissemination of the data, thus, they have not been made publicly available.
