# Supplementary material for: Economic burden of patients with post-surgical chronic and transient hypoparathyroidism in the United States examined using insurance claims data
Source: Orphanet J Rare Dis. 2024 Apr 18;19:164. doi: 10.1186/s13023-024-03155-4 (PMC11025287; doi:10.1186/s13023-024-03155-4)
Supplement: Supplementary file 1 — Supplementary Material 1 [file 13023_2024_3155_MOESM1_ESM.docx]

**SUPPLEMENTARY MATERIALS**

1. Supplementary Figure 1. Study Period
2. Supplementary Figure 2. Chronic HP Patient Selection Flowchart
3. Supplementary Figure 3. Transient HP Patient Selection Flowchart
4. Supplementary Table 1. Diagnosis and Procedure Codes for Selection Criteria

**Supplementary Figure 1: Study Period**

*^a^cHP Index Date: defined as the date of the first qualifying HP diagnosis claim; tHP Index Date: defined as the date of the last HP diagnosis claim.*

*cHP: Chronic Hypoparathyroidism; HP: Hypoparathyroidism; tHP: Transient Hypoparathyroidism*

**Supplementary Figure 2: Patient selection flowchart for Chronic HP cohort**

**
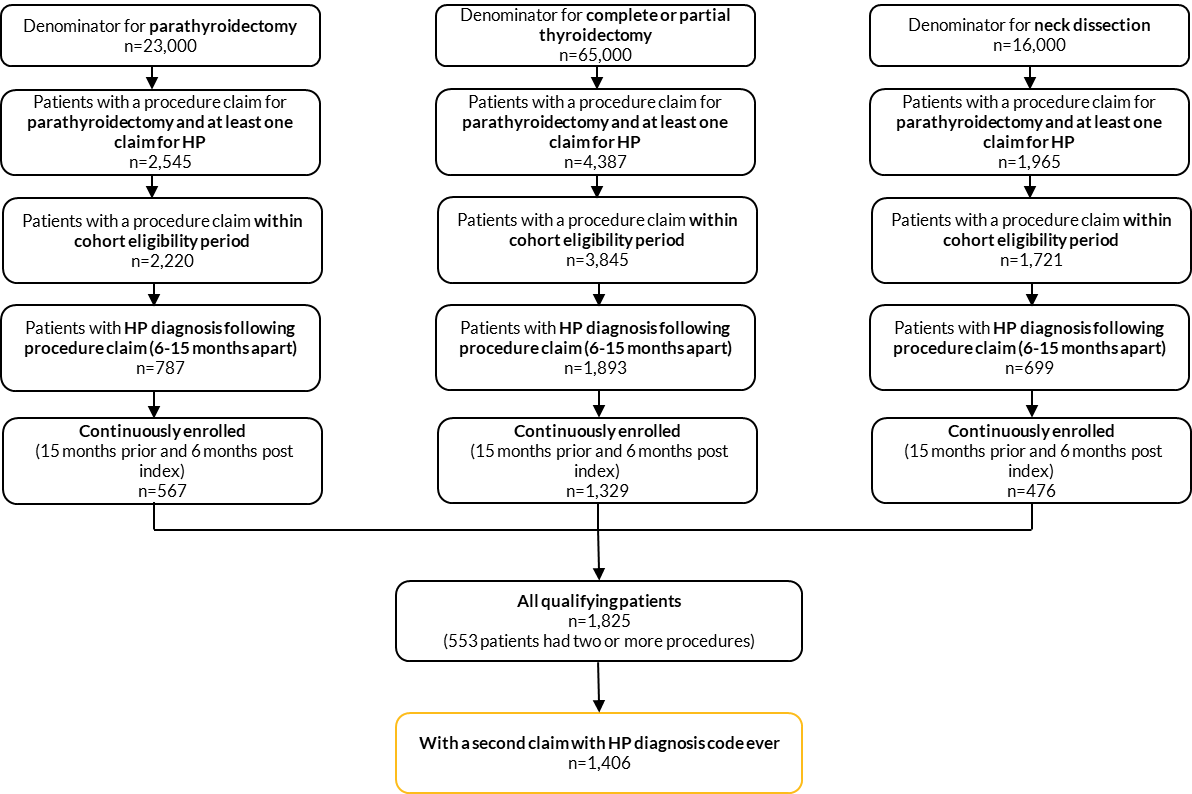
**

*HP: Hypoparathyroidism*

**Supplementary Figure 3: Patient selection flowchart for Transient HP cohort**

**
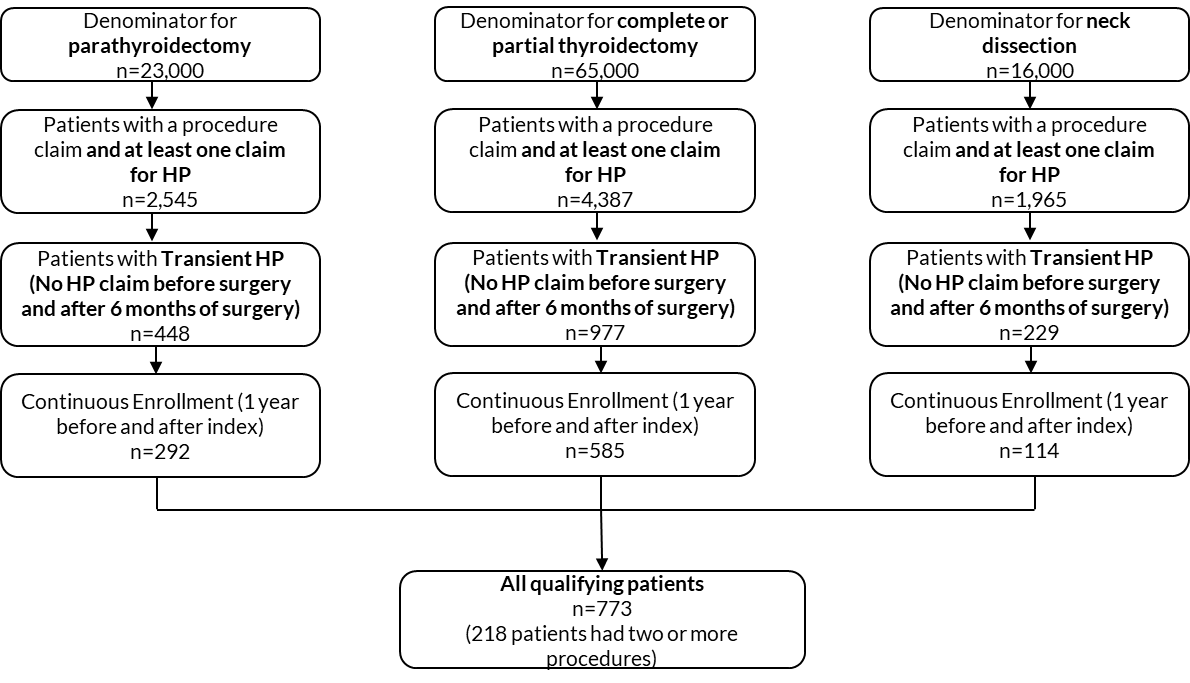
**

*HP: Hypoparathyroidism*

**Supplementary Table 1: Diagnosis and Procedure Codes for Selection Criteria**

| Diagnosis/Procedure | ICD 9 | ICD 10 | CPT |
| --- | --- | --- | --- |
| Hypoparathyroidism | 252.1 | E20.0, E20.8, E20.9, E89.2 | Not applicable |
| Parathyroidectomy | 068, 0681, 0689 | 0GTR0ZZ, 0GTR4ZZ, 0G5R0ZZ, 0G5R3ZZ, 0G5R4ZZ, 0GBR0ZZ, 0GBR3ZZ, 0GBR4ZZ | 60500, 60502 |
| Complete or partial Thyroidectomy | 064, 062, 063, 063, 0639, 065, 0650, 0651, 0652 | 0GTG0ZZ, 0GTG4ZZ, 0GTH0ZZ, 0GTH4ZZ, 0G5K0ZZ, 0G5K3ZZ, 0G5K4ZZ, 0GBG0ZZ, 0GBG3ZZ, 0GBG4ZZ, 0GBH0ZZ, 0GBH3ZZ, 0GBH4ZZ, 0GTK0ZZ, 0GTK4ZZ, 0GBJ0ZZ, 0GBJ3ZZ, 0GBJ4ZZ | 60220, 60225, 60240, 60252, 60254, 60270  60271, 60212, 60210 |
| Neck Dissection | 4040, 4041, 4042 | 07T10ZZ, 07T14ZZ, 07T20ZZ, 07T24ZZ | 38724, 38720 |
